# Microbial Debromination of Polybrominated Diphenyl Ethers by *Dehalococcoides*-Containing Enrichment Culture

**DOI:** 10.3389/fmicb.2021.806795

**Published:** 2022-02-17

**Authors:** Siyan Zhao, Siyan Fan, Yide He, Yongjun Zhang

**Affiliations:** School of Environmental Science and Engineering, Nanjing Tech University, Nanjing, China

**Keywords:** microbial degradation, reductive debromination, *Dehalococcoides*, bioremediation, polybrominated diphenyl ether (PBDEs)

## Abstract

Polybrominated diphenyl ethers (PBDEs), commonly used as flame retardants in a wide variety of consumer products, are emerging persistent pollutants and ubiquitously distributed in the environment. The lack of proper bacterial populations to detoxify these recalcitrant pollutants, in particular of higher brominated congeners, has confounded the attempts to bioremediate PBDE-contaminated sites. In this study, we report a *Dehalococcoides*-containing enrichment culture, PB, which completely debrominates 0.44 μM tetra-brominated diphenyl ether (BDE) 47 to diphenyl ether within 25 days (0.07 μM Br^–^/day) and extensively debrominates 62.4 ± 4.5% of 0.34 μM hepta-BDE 183 (0.006 μM Br^–^/day) with a predominant generation of penta- through tri-BDEs as well as small amounts of diphenyl ether within 120 days. Later, a marked acceleration rate (0.021 μM Br^–^/day) and more extensive debromination (87.7 ± 2.1%) of 0.38 μM hepta-BDE 183 was observed in the presence of 0.44 μM tetra-BDE 47, which is achieved *via* the faster growth rate of responsible bacterial populations on lower BDE-47 and debromination by expressed BDE-47 reductive dehalogenases. Therefore, the PB enrichment culture can serve as a potential candidate for *in situ* PBDE bioremediation since both BDE-47 and BDE-183 are dominant and representative BDE congeners and often coexist in contaminated sites.

## Introduction

Polybrominated diphenyl ethers (PBDEs) are a class of commonly used flame retardants and widely added in a variety of products, from construction materials, electrical and electronic equipment to household products and textiles. Their toxicity and persistence have drawn great public concerns that the commercially manufactured PBDEs [deca-brominated diphenyl ether (BDE) mixture, octa-BDE mixture, and penta-BDE mixture] were listed as persistent organic pollutants by the Stockholm Convention, as well as their use were banned or gradually phasing out (UNEP, 2009). Though restrictions and bans on manufacture and usage have significantly reduced the amounts of new BDEs introduced into the environment, these legislations have no effect on the release of BDEs from existing products or from recycled materials containing BDEs. For example, unregulated recycling of electronics and electrical components has recently become a major source of environmental contamination in developing countries, particularly in Southeast Asia and China (Luo et al., 2009; Li et al., 2016; Matsukami et al., 2017; Lu et al., 2021).

Products containing PBDEs are dumped into landfills from which they may leach into nearby sediments and soils and eventually partition into anaerobic or anoxic environmental compartments due to their high hydrophobicity and become a persistent emission source (O’Driscoll et al., 2016). As anaerobic and anoxic benthic sediments and soils are major sinks and environmental reservoirs for PBDEs, anoxic dehalogenation by microorganisms becomes an important route for eliminating PBDEs in such an environment. Compared with the lower BDE congeners (with 1–4 bromines), higher BDE congeners (with 5–10 bromines) are usually of lower bioavailability and are more resistant to microbial degradation (Palm et al., 2002). Currently, both microcosms established from a versatile environment and isolated microorganisms have shown slower and less extensive debromination on deca- through hexa-BDEs rather than penta- and tetra-BDEs (Lee and He, 2010). So far, only partial and incomplete debromination of deca- through hexa-BDEs (Robrock et al., 2008) and a limited number of microorganisms capable of complete debromination of penta- and tetra-BDEs have been reported (Ding et al., 2017; Zhao et al., 2021). Therefore, cultures that can more efficiently debrominate PBDEs and further be applied in *in situ* warrant further exploration.

The supplement of auxiliary substrates, such as other organohalides (i.e., trichloroethene, chlorophenols) or organics (i.e., lactate) has been proven as an effective strategy in lab-scale studies (Robrock et al., 2008; Lee and He, 2010). However, such co-contaminations by or priming of other organohalide contaminants, and the biostimulation of additional nutrients, seem less practical due to the introduction of new toxins and less economic efficiency in practical applications. The exploration of practical auxiliary substrates to stimulate the debromination process is likely a feasible solution of the elimination of higher BDEs in contaminated sites. Lower BDEs generally coexist with higher BDEs in the environment, either from direct release from multiple BDE-containing products or *via* the transformation from the higher BDEs. Being organohalides of higher bioavailability and widely coexisting with higher BDEs, lower BDEs seem to be a potential candidate as an auxiliary substrate for more accelerated and extensive debromination of higher BDEs. However, the co-existence of lower and higher BDE congeners may also lead to the inhibition on the microbial debromination of each other (Yan et al., 2018; Wang et al., 2021). Therefore, whether more accelerated and extensive debromination of higher BDEs could be achieved *via* the stimulated growth of PBDE-debrominating populations and the inhibition release after the removal of lower BDEs from the environment remains unclear, which warrants further validation and exploration.

Hepta-BDE 183, the most dominant congener in and used as the detection indicator for commercial octa-BDE mixtures, and BDE-47, one of the most commonly and dominantly detected types in commercial penta-BDE mixtures, were selected as the representative higher and lower BDE ortholog congeners to screen for the PBDE-debrominating cultures as the candidate for *in situ* bioremediation and explore the strategies to improve the debromination efficiency. In this study, we enriched a culture that exhibited complete debromination of BDE-47 and extensive debromination of BDE-183. Through microbial community and molecular analysis, the functional bacterial population was identified. Later, the strategy of using lower BDEs as the auxiliary substrate for improved debromination of higher BDEs, as well as the involved mechanisms, was investigated by spiking both BDE-47 and BDE-183 in this enrichment culture.

## Materials and Methods

### Chemicals

Individual congeners (hepta-BDE 183 and tetra-BDE 47) were purchased from Agilent Technologies, Inc. (Santa Clara, CA, United States) at 5,000 ppm, dissolved in isooctane (2,2,4-trimethylpentane). PBDE congener mixtures comprising 39 congeners (from hexa- to mono-BDEs at concentrations from 100 to 250 mg/L) at purities above 98% were purchased from Cambridge Isotope Laboratories, Inc. (Andover, MA, United States), and were used as standards for the method development of detection and quantification. Other BDE congeners were purchased from AccuStandard (New Haven, CT, United States).

### Microcosm Setup, Culture Enrichment, and Growth Conditions

Microcosms with PBDE-debrominating activity were initially established by the amendment of 10% (w/v) soils from an e-waste recycling site to a 60 ml serum bottle containing 30 ml bicarbonate buffered, defined DCB1 minimal salts medium (He et al., 2007). Lactate (10 mM) was added as carbon source and electron donor, as well as BDE-47 (∼0.47 μM) was spiked as electron acceptor. Abiotic controls without sediments or with autoclaved soils/sediments were established. All microcosms were incubated in the dark at 30°C.

The microcosm showing complete debromination of BDE-47 to diphenyl ether was selected for further exploration. After three consecutive transfers, a sediment-free culture, designed PB, was obtained. The sediment-free culture was further enriched by three batches of 10^–1^–10^–5^ serial dilution using acetate (10 mM) as carbon source, hydrogen (0.33 atm) as electron donor, and BDE-47 (∼0.47 μM) as electron acceptor. Finally, an enrichment culture with the same BDE-47 debromination profile as the original microcosm was attained. The PBDE debromination kinetic studies by the PB enrichment culture were performed in 160 serum bottles containing 100 ml medium as described above, using acetate (10 mM) as carbon source, hydrogen (0.33 atm) as electron donor, and BDE congeners (i.e., BDE-47 and BDE-183) as indicated in the Results section. All the kinetics studies were at a 5% inoculum size and were incubated in the dark without shaking at 30°C in triplicates. Controls were established in DCB1 medium with the spike of BDE congener as indicated, in either without the inoculation of the PB enrichment culture or with the autoclaved PB enrichment culture.

### Chemical Analysis

BDEs were detected and quantified using an Agilent gas chromatograph-mass spectrometer (GC6890-MSD5975) equipped with an Rxi-5 ms column (15 m × 0.25 mm × 0.25 μm; Restek, Bellefonte, PA, United States) as previously described (Lee and He, 2010). Briefly, BDEs in cultures were extracted with isooctane (1:1, v/v) using deca-bromobiphenyl (DBB) as an internal standard. The oven temperature was initially set at 110°C, increased to 310°C at a rate of 15°C/min, and held at 310°C for 3 min. Calibration curves of the spiked congeners (BDE-47, BDE-183) were quantified using serum bottles containing defined DCB1 medium with defined amounts. Considering that some degradative metabolites were commercially unavailable, the standard curves of the degradative metabolites were established using the average peak area of congeners in the same homolog group. A total of six standard curves (including penta-, tetra-, tri-, di-, mono-diphenyl ether, and diphenyl ether) were established; the standard deviation of each curve was within 20%. The bromine removal amount was calculated by the deduction of the bromine amounts of all detected PBDE congeners at a given time from the bromine amounts on day 0.

### Molecular Analysis

The 16S rRNA-based microbial community of the PB enrichment culture was analyzed using the 515F and 906R primers on an Illumina MiSeq platform. The DNA was extracted from a BDE-47-spiked PB enrichment culture using Qiagen Blood & Tissue kit (QIAGEN, Hilden, Germany) according to instructions. The microbial community was analyzed using QIIME2 v2019.1.0 (Bolyen et al., 2019). Paired-end reads were joined, quality-filtered, dereplicated, and *de novo* clustered at 97% identity using the Vsearch Plugin (Rognes et al., 2016). Chimeric operational taxonomic units (OTUs) were identified (QIIME2 Vsearch Uchime-*DeNovo* Plugin) and removed, leaving 65,940 sequences in 52 OTUs, with a Pielou evenness calculated at 0.206. Default parameters were used unless stated otherwise. To monitor the growth of the *functional bacterial* populations during BDE debromination, quantitative real-time PCR (qPCR) targeting *Dehalococcoides* 16S rRNA with primers Dhc-qF2/Dhc-qR (Freeborn et al., 2005). Plasmids carrying target genes serving as standards for qPCR were constructed using pGEM-T vector systems kit (Promega, Madison, WI, United States) and extracted using QIAprep Spin Minprep kit (QIAGEN, GmbH, Hilden, Germany). The calibration curves were established using serial dilutions of known plasmid DNA concentrations ranging from 10^2^ to 10^8^ per reaction.

### Enzymatic Assays

Enzymatic assays were performed to investigate the debrominating activity of hepta-BDE-183 by the crude cell extracts of 200 ml PB enrichment culture grown on 0.47 μM tetra-BDE 47. Cells were harvested by centrifugation (12,000 rpm, 10 min, 4°C) and resuspended in degassed Tris-HCl buffer (100 mM, pH 7.0). Crude extracts were obtained by disrupting cells using a VCX 130 sonicator (130 W; 20% duty cycle; 3 min). *In vitro* assays were carried out in 4 ml vials containing 2 ml assay solution [2 mM methyl viologen; 1.5 mM titanium (III) citrate; 100 mM Tris-HCl buffer (pH 7.0)] inside an anaerobic chamber as previously described (Adrian et al., 2007). Hepta-BDE 183 (0.4 μM) was added to duplicate 4 ml vials containing the assay solution. The test was initiated by addition of cell extracts, and the mixtures were incubated at 30°C for 72 h prior to analysis.

### Sequence Data Deposit

The Illumina sequencing results of the PB enrichment culture were deposited to the NCBI under BioProject, PRJNA774086.

## Results

### Debromination of Brominated Diphenyl Ethers by PB Enrichment Culture

**FIGURE 1:**
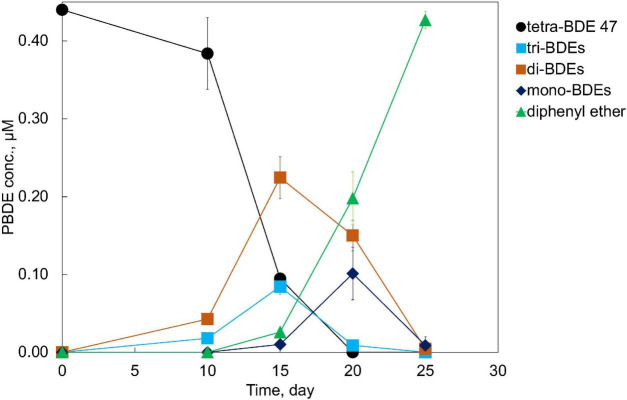
Debromination of tetra-BDE 47 by PB enrichment culture.

**FIGURE 2:**
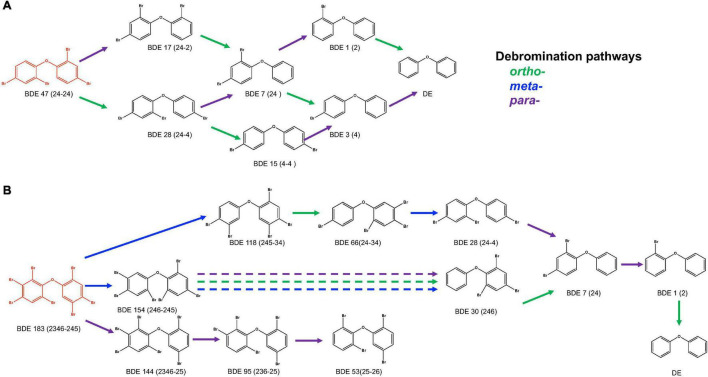
Debromination pathways of tetra-BDE 47 and hepta-BDE 183 by PB enrichment culture. DE, diphenyl ether.

An anaerobic mixed culture (designated culture PB) to perform a microbial debromination of tetra-BDE 47, a dominant congener among lower BDE ortholog groups present in the environment, was enriched from an e-waste-contaminated site. After three batches of serial dilution (10^–1^–10^–5^) in a mineral-salts medium amended with acetate (10 mM) as a carbon source, hydrogen (0.3 atm) as an electron donor, and tetra-BDE 47 as the sole electron acceptor, this mixed culture was further enriched. This PB enrichment culture completely debrominated 0.44 μM tetra-BDE 47 *via* stepwise debromination to a non-toxic end product, diphenyl ether, at an averaged debromination rate of 0.07 μM Br^–^ removal/day within 25 days (Figure 1). During the debromination process, several metabolic intermediates were detected, including two tri-BDEs (congener 17, 28), two di-BDEs (congener 7, 15), and two mono-BDEs (congener 1,3) (Figure 2A). Both *ortho*- and *para*-bromine were attacked for substitutions, and no preferential removal pathway was observed.

**FIGURE 3:**
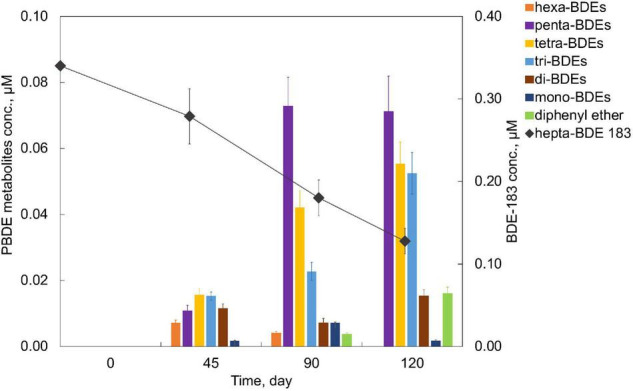
Debromination of hepta-BDE 183 by PB enrichment culture.

An extensive debromination of one of the representative high BDEs, hepta-BDE 183, was exhibited by the PB enrichment culture. Within 120 days of cultivation, 62.4 ± 4.5% of 0.34 μM hepta-BDE 183 was debrominated at a debromination rate of 0.006 μM Br^–^ removal/day (Figure 3). Penta- through mono-BDEs as well as diphenyl ether were produced as end products with an average decrease of bromine/diphenyl ether from 7 to 4.89 ± 0.37, with a 30.14 ± 5.3% removal of the total bromine. Among these metabolites, penta-BDEs were the most dominant accumulated congener groups of 20.9 ± 3.1%, followed by tetra-BDEs and tri-BDEs (16.3 ± 2.0 and 15.4 ± 1.9%, respectively), though in a small amount of 4.7 ± 0.6%, diphenyl ether was also detected. During the debromination process, a metabolite mixture, including two hexa-BDEs (congener 154, 144), three penta-BDEs (congener 118, 103, 95), two tetra-BDEs (congener 66, 53), two tri-BDEs (congener 30, 28), di-BDE 7, and mono-BDE 1, were detected (Figure 2B). Though all *ortho*-, *meta*-, and *para*-bromine removals were observed, the latter two pathways were preferred during the initiation of the debromination (from hepta-BDE to hexa-BDEs).

### Identification of Polybrominated Diphenyl Ether-Debrominating Bacterial Populations in PB Enrichment Culture

**FIGURE 4:**
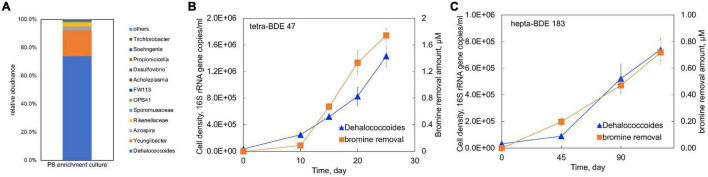
**(A)** Microbial community structure of the PB enrichment culture determined by 16S rRNA gene amplicon sequencing; growth of *Dehalococcoides* coupled with **(B)** tetra-BDE 47 and **(C)** hepta-BDE 183 debromination.

The microbial community of the PB enrichment culture grown on tetra-BDE 47 was elucidated *via* an Illumina sequencing of the V4 and V5 regions of the 16S rRNA gene. Four bacterial taxonomic groups (*Dehalococcoides, Youngiibacter, Azospira*, and *Rikenellaceae*) dominated the PB enrichment culture, accounting for more than 98.0% of the entire community (Figure 4A), of which *Dehalococcoides* (74% of the community) is the only taxonomic group known to dehalogenate organohalide compounds. The cell abundance of *Dehalococcoides* in the PB enrichment culture increased 40.9-fold during debromination, from 3.50 ± 0.42 × 10^4^ to 1.43 ± 0.16 × 10^6^ 16S rRNA gene copies/ml (Figure 4B). The increase in *Dehalococcoides* abundance had a positive linear correlation with bromine removal (*R*^2^ = 0.94) with a calculated growth yield of 8.21 × 10^7^ cells/μmoles Br^–^ removed. Similarly, the debromination of hepta-BDE 183 was also coupled with the growth of *Dehalococcoides* (Figure 4C) (*R*^2^ = 0.96) at an estimated growth yield of 1.03 × 10^8^ cells/μmol Br^–^ removed. Therefore, *Dehalococcoides* was identified as the essential player in PBDE debromination by the PB enrichment culture.

### Enhanced Debromination of Hepta-BDE 183 by Concurrence of Tetra-BDE 47 by PB Enrichment Culture

**FIGURE 5:**
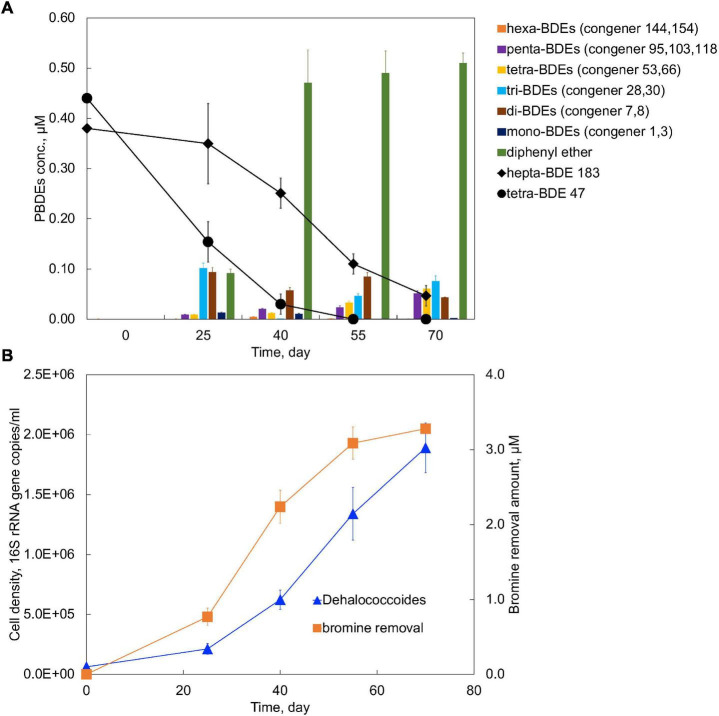
**(A)** Debromination kinetics and **(B)** growth of *Dehalococcoides* in the PB enrichment culture on the co-occurrence of tetra-BDE 47 and hepta-BDE 183.

Lower brominated diphenyl ethers often coexist with higher brominated ones due to direct disposal, as well as transformation from either abiotic or microbial degradation from higher brominated ones. The synergistic effects of the coexistence of both higher and lower brominated congener groups, i.e., hepta-BDE 183 and tetra-BDE 47, on the performance of microbial degradation by the PB enrichment culture were investigated. In total, 87.7 ± 2.1% removal of 0.38 μM hepta-BDE 183, producing hexa- through diphenyl ether, and a complete removal of tetra-BDE 47 was achieved within 70 days (Figure 5A). During this debromination process, the average debromination rate of both hepta-BDE 183 and tetra-BDE 47 was 0.047 μM Br^–^ removal/day, which was 33% lower than that of the spike of tetra-BDE 47 solely (0.07 μM Br^–^ removal/day), but 7.83 times faster than that of the spike of hepta-BDE 183 solely (0.006 μM Br^–^ removal/day).

Notably, a marked acceleration of hepta-BDE 183 debromination was achieved while co-existing with tetra-BDE 47, with an estimated debromination rate of 0.021 μM Br^–^ removal/day, 3.62 times faster than that of sole spike of hepta-BDE 183. Meanwhile, hepta-BDE-183 was debrominated more extensively with an average decrease of bromine/diphenyl ether from 7.0 to 3.00 ± 0.21 with a 57.14 ± 3.0% removal of the total bromines, compared with to 4.89 ± 0.37 with a 30.14 ± 5.3% total bromine removal for its sole spike. Diphenyl ether was detected as the most dominant end product of 26.3 ± 2.3% (with the deduction of diphenyl ether produced from tetra-BDE 47), followed by tri-BDEs of 19.8 ± 3.3% and tetra-BDEs of 16.0 ± 2.1%. Under these two circumstances, an obvious shift of the debrominating metabolites’ profile was observed that the most dominant BDEs’ congener group was changed from penta-BDEs to tri-BDEs, a less brominated and less toxic group. All three *ortho*- *meta*-, and *para*- removal pathways were found during the debromination of concurrence of hepta-BDE 183 and tetra-BDE 47 by the PB enrichment culture, but with the detection of additional metabolic intermediates, i.e., penta-BDE 95 and di-BDE 8, compared with the sole spike of either hepta-BDE 183 or tetra-BDE 47.

A noticeable deceleration of tetra-BDE 47 debromination process was observed that 55 days were taken to debrominate 0.44 μM tetra-BDE 47 at its co-existence with hepta-BDE 183, compared with 25 days for tetra-BDE 47 at the same amount solely (Figure 5A). Within these 55 days, the average debromination rate of all BDEs (including both hepta-183 and tetra-BDE 47, together) was estimated at 0.056 Br^–^ removal/day, 20% slower than that of sole spike of tetra-BDE 47.

### Mechanisms of Enhanced Hepta-BDE 183 Debromination at Concurrence of Tetra-BDE 47

The presence of tetra-BDE 47 provided the PB enrichment culture a more accessible electron acceptor compared with hepta-BDE 183, which was supported by a faster debromination rate and shorter debromination process. The energy gained from the debromination process of tetra-BDE 47 supported a faster growth rate of *Dehalococcoides* at a doubling time of 14.18 days (Figure 5B), than that on hepta-BDE 183 solely of 26.72 days. This faster growth rate of BDE debrominating population directly yielded a higher cell density within the same period, resulting in a higher debrominating activity.

*In vitro* enzymatic assays by the crude cell extracts of the PB enrichment culture grown on tetra-BDE 47 also showed a debrominating activity on hepta-BDE 183 (Table 1). About 12.1 ± 4.2% of hepta-BDE 183 was debrominated with an average decrease of bromine/diphenyl ether from 7 to 6.79 ± 0.09, producing penta-BDEs (congener 95 and 103, 9.0 ± 3.2%) and hexa-BDEs (congener 154 and 144, 3.1 ± 1.7%). The debrominating profiles were not significantly shifted even at a higher dose concentration of crude cell extracts and longer incubation time in enzymatic assays. Compared with hepta-BDE 183 debromination *in vivo*, distinct debrominating profiles were observed, with a much less debromination extent and different metabolic intermediates. Together with the similar phenomenon (i.e., more extensive debromination and generation of new metabolic intermediates) observed in *in vivo* PB enrichment cultures with the co-occurrence of tetra-BDE 47, these observed distinct debrominating profiles suggested that the debromination of hepta-BDE 183 and tetra-BDE 47 were not likely mediated by the same reductive dehalogenases, which probably contributed to the more extensive debromination of hepta-BDE 183 at an additional 27% bromine removal (57.14 ± 3.0 vs. 30.14 ± 5.3%) of hepta-BDE 183 on the concurrence of tetra-BDE 47.

**TABLE 1:** Debromination metabolites from hepta-BDE 183 by PB enrichment culture after cultivation (*in vivo*) with BDE 183 solely and co-spike of BDE 47 and 183, as well in enzymatic assays of crude cell extracts (*in vitro*) of cultures spiked with BDE 47.

		*In vivo*		*In vitro*
IUPAC number	Compound/substituents	183	47 and 183	183
DE		4.7%	26.3%	
Mono-1	2	0.5%	0.5%	
Di-7	24	4.5%	1.2%	
Di-8	2–4		10.2%	
Tri-30	246	3.8%	15.2%	
Tri-28	24–4	11.7%	4.6%	
Tetra 53	25–26	5.8%	6.2%	
Tetra-66	24–34	10.4%	9.80%	
Penta-95	236–25	11.7%	13.50%	7.8%
Penta-103	246–25			1.2%
Penta-118	245–34	9.2%		
Hexa-154	245–246			2.1%
Hexa-144	2346–25			1.0%
Hepta-183	2346–245	37.6%	12.30%	87.9%

*IUPAC = International union of pure and applied chemistry.*

## Discussion

In this study, an enriched-culture PB was found to possess a unique debrominating capability toward both higher and lower BDEs. This identified that the PB enrichment culture exhibited a rapid and complete debromination of tetra-BDE 47 (to diphenyl ether at 0.44 μM within 25 days). During the debromination process, the multiple metabolic intermediates of different PBDE ortholog groups were observed, suggesting expanded debrominating capabilities of lower brominated congeners by this PB enrichment culture. Meanwhile, this PB enrichment culture exhibited a significant removal of higher BDEs, with 62.4 ± 4.5% removal of hepta-BDE 183 within 4 months, and noticeably extensive bromine removal, predominantly producing penta- through tri-BDE congeners and generating diphenyl ether in trace amounts from hepta-BDE 183. Comparatively, a less extensive debromination of hepta-BDE 183 has been observed in previously reported microcosms or cultures, the debromination of which often ceased at hexa- through tetra-BDEs (Lee and He, 2010). For example, hexa-BDEs were the most dominant debrominating products in investigation-involved soils and sediments from 28 locations (Lee and He, 2010), though more extensive debromination (i.e., debrominating a higher extent of higher BDEs and generating lesser-brominated congeners) could be achieved *via* the supplement of auxiliary substrates in these previously reported microcosms and cultures, such as other organohalide contaminants (e.g., trichloroethene or chlorophenols) or organic nutrients (e.g., lactate) (He et al., 2006; Robrock et al., 2008; Ding et al., 2013; Stiborova et al., 2015). Even without the supplement of auxiliary substrates, the PB enrichment culture still showed a more competitive debrominating performance that even lower-brominated congeners of tri- and di-BDEs as well as diphenyl ether were detected, thus making the PB enrichment culture a strong candidate for the *in situ* treatment of BDE contaminants.

The versatile dehalogenating capabilities on both lower and higher BDEs make the PB enrichment culture even more competitive *in situ* to cope with PBDE-contaminated sites due to the often coexistence of different BDE congener groups in the environment. By the coexistence of lower BDE-47, an enhanced removal of higher BDE-183 was achieved, showing (i) 7.83 times faster debrominating rate, (ii) increased the debromination extent of BDE-183 by 25.2%, and (iii) more extensive bromine removal by 27% with predominant production of tri-BDEs instead of penta- and tetra-BDEs, which are the most toxic BDE ortholog groups. This enhancement greatly shortened the debromination period and achieved more extensive debromination of higher BDE-183. The dominantly produced tri-BDEs are less toxic and tend to be more readily degraded by microorganisms in the environment (ATSDR, 2017; Komolafe et al., 2021), thus likely reducing the public health risks. Generally, the supplement of the auxiliary substrates of more readily utilized electron acceptors, such as other organohalide pollutants (e.g., trichloroethene) or priming organic nutrients (e.g., lactate) have shown an obvious enhancement of BDE debromination in lab-scale studies (Robrock et al., 2008; Lee and He, 2010; Ding et al., 2013). However, the priming of an equivalent or even more toxic pollutants makes the strategy difficult to be applied in field. Yet, the finding in this study can relieve the doubt that the lower BDE is a ubiquitously distributed co-contaminant with higher BDEs, either from degradation *via* different pathways (e.g., chemical, physical, or biological) or direct release from disposal. The indigenous lower BDE on site could possibly serve as auxiliary substrates for the bioaugmented cultures that could both utilize lower and higher BDE congeners, such as the PB enrichment culture, thus participating in the treatment of PBDE contaminants.

Compared with lower BDE congener groups, higher BDEs are more persistent and recalcitrant for biodegradation in the environment. Thus far, a complete debromination of lower BDE congeners (such as penta-BDE 99, 100, and tetra-BDE 47) has been found in the bacterial populations of *Dehalococcoides* (Ding et al., 2017; Zhao et al., 2021), yet difficulties still lie in the discovery of proper bacterial populations to completely detoxify higher BDEs; only slow and partial biodegradation, which often leads to the accumulation of even more toxic congener groups, has been reported (Zhao et al., 2018). In the PB enrichment culture, multiple RDases are suggested to catalyze higher and lower BDEs (i.e., BDE-183 and BDE-47), which is consistent with previous studies that multiple RDases were identified in the debromination of penta-BDEs (Ding et al., 2017; Zhao et al., 2021). Taken together, to improve the removal effectiveness and efficiency of higher BDEs in practical application, we may consider the strategy of bio-augmenting cultures with a broader dehalogenating range of PBDE congeners, which could be achieved by integrating multiple bacterial populations with more versatile dehalogenating capabilities on different PBDE congener orthologs.

## Data Availability Statement

The datasets presented in this study can be found in online repositories. The names of the repository/repositories and accession number(s) can be found in the article/supplementary material.

## Author Contributions

SZ and YZ contributed to conception and design of the study. SZ, SF, and YH performed the experiment and statistical analysis. SZ, SF, and YZ wrote the manuscript. All authors contributed to manuscript revision, read, and approved the submitted version.

## Conflict of Interest

The authors declare that the research was conducted in the absence of any commercial or financial relationships that could be construed as a potential conflict of interest.

## Publisher’s Note

All claims expressed in this article are solely those of the authors and do not necessarily represent those of their affiliated organizations, or those of the publisher, the editors and the reviewers. Any product that may be evaluated in this article, or claim that may be made by its manufacturer, is not guaranteed or endorsed by the publisher.

## References

[B1] Adrian L. Rahnenführer J. Gobom J. Hölscher T. (2007). Identification of a chlorobenzene reductive dehalogenase in *Dehalococcoides* sp. strain CBDB1. *Appl. Environ. Microbiol.* 73 7717–7724. 10.1128/AEM.01649-07 17933933 PMC2168065

[B2] ATSDR (2017). *Toxicological Profile for Polybrominated Diphenyl Ethers (PBDEs), Public Health Service, Department of Health and Human Services.* (Atlanta, GA: Agency for Toxic Substances and Disease Registry). 37262200

[B3] Bolyen E. Rideout J. R. Dillon M. R. Bokulich N. A. Abnet C. C. Al-Ghalith G. A. (2019). Reproducible, interactive, scalable and extensible microbiome data science using QIIME 2. *Nat. Biotechnol.* 37 852–857. 31341288 10.1038/s41587-019-0209-9 PMC7015180

[B4] Ding C. Chow W. L. He J. (2013). Isolation of *Acetobacterium* sp. strain AG, which reductively debrominates octa- and pentabrominated diphenyl ether technical mixtures. *Appl. Environ. Microbiol.* 79 1110–1117. 10.1128/AEM.02919-12 23204415 PMC3568622

[B5] Ding C. Rogers M. J. Yang K. L. He J. Z. (2017). Loss of the ssrA genome island led to partial debromination in the PBDE respiring *Dehalococcoides mccartyi* strain GY50. *Environ. Microbiol.* 19 2906–2915. 10.1111/1462-2920.13817 28618081

[B6] Freeborn R. A. West K. A. Bhupathiraju V. K. Chauhan S. Rahm B. G. Richardson R. E. (2005). Phylogenetic analysis of TCE-dechlorinating consortia enriched on a variety of electron donors. *Environ. Sci. Technol.* 39 8358–8368. 10.1021/es048003p 16294874

[B7] He J. Holmes V. F. Lee P. K. H. Alvarez-Cohen L. (2007). Influence of vitamin B12 and cocultures on the growth of *Dehalococcoides* isolates in defined medium. *Appl. Environ. Microbiol.* 73 2847–2853. 10.1128/AEM.02574-06 17337553 PMC1892872

[B8] He J. Robrock K. R. Alvarez-Cohen L. (2006). Microbial reductive debromination of polybrominated diphenyl ethers (PBDEs). *Environ. Sci. Technol.* 40 4429–4434. 10.1021/es052508d 16903281

[B9] Komolafe O. Mrozik W. Dolfing J. Acharya K. Vassalle L. Mota C. R. (2021). Fate of four different classes of chemicals under aerobic and anaerobic conditions in biological wastewater treatment. *Front. Environ. Sci.* 9:700245. 10.3389/fenvs.2021.700245

[B10] Lee L. K. He J. (2010). Reductive debromination of polybrominated diphenyl ethers by anaerobic bacteria from soils and sediments. *Appl. Environ. Microbiol.* 76 794–802. 10.1128/AEM.01872-09 20008168 PMC2812996

[B11] Li W.-L. Ma W.-L. Jia H.-L. Hong W.-J. Moon H.-B. Nakata H. (2016). Polybrominated diphenyl ethers (PBDEs) in surface soils across five Asian countries: levels, spatial distribution, and source contribution. *Environ. Sci. Technol.* 50 12779–12788. 10.1021/acs.est.6b04046 27775342

[B12] Lu Q. Liang Y. Fang W. Guan K. L. Huang C. Qi X. (2021). Spatial distribution, bioconversion and ecological risk of PCBs and PBDEs in the surface sediment of contaminated urban rivers: a nationwide study in China. *Environ. Sci. Technol.* 55 9579–9590. 10.1021/acs.est.1c01095 33852286

[B13] Luo Y. Luo X.-J. Lin Z. Chen S.-J. Liu J. Mai B.-X. (2009). Polybrominated diphenyl ethers in road and farmland soils from an e-waste recycling region in Southern China: concentrations, source profiles, and potential dispersion and deposition. *Sci. Total Environ.* 407 1105–1113. 10.1016/j.scitotenv.2008.10.044 19019410

[B14] Matsukami H. Suzuki G. Someya M. Uchida N. Tue N. M. Viet P. H. (2017). Concentrations of polybrominated diphenyl ethers and alternative flame retardants in surface soils and river sediments from an electronic waste-processing area in northern Vietnam, 2012–2014. *Chemosphere* 167 291–299. 10.1016/j.chemosphere.2016.09.147 27728888

[B15] O’Driscoll K. Robinson J. Chiang W.-S. Chen Y.-Y. Kao R.-C. Doherty R. (2016). The environmental fate of polybrominated diphenyl ethers (PBDEs) in western Taiwan and coastal waters: evaluation with a fugacity-based model. *Environ. Sci. Pollut. Res.* 23 13222–13234. 10.1007/s11356-016-6428-4 27023809 PMC4912977

[B16] Palm A. Cousins I. T. Mackay D. Tysklind M. Metcalfe C. Alaee M. (2002). Assessing the environmental fate of chemicals of emerging concern: a case study of the polybrominated diphenyl ethers. *Environ. Pollut.* 117 195–213. 10.1016/s0269-7491(01)00276-7 11916035

[B17] Robrock K. R. Korytar P. Alvarez-Cohen L. (2008). Pathways for the anaerobic microbial debromination of polybrominated diphenyl ethers. *Environ. Sci. Technol.* 42 2845–2852. 10.1021/es0720917 18497133

[B18] Rognes T. Flouri T. Nichols B. Quince C. Mahé F. (2016). VSEARCH: a versatile open source tool for metagenomics. *PeerJ* 4:e2584. 10.7717/peerj.2584 27781170 PMC5075697

[B19] Stiborova H. Vrkoslavova J. Pulkrabova J. Poustka J. Hajslova J. Demnerova K. (2015). Dynamics of brominated flame retardants removal in contaminated wastewater sewage sludge under anaerobic conditions. *Sci. Total Environ.* 533 439–445. 10.1016/j.scitotenv.2015.06.131 26179781

[B20] UNEP (2009). *Stockholm Convention on Persistent Organic Pollutans (POPs).* (Geneva: United Nations Environment Programme).

[B21] Wang G. Jiang N. Liu Y. Wang X. Liu Y. Jiao D. (2021). Competitive microbial degradation among PBDE congeners in anaerobic wetland sediments: implication by multiple-line evidences including compound-specific stable isotope analysis. *J. Hazard. Mater.* 412:125233. 10.1016/j.jhazmat.2021.125233 33513555

[B22] Yan Y. Li Y. Ma M. Ma W. Cheng X. Xu K. (2018). Effects of coexisting BDE-47 on the migration and biodegradation of BDE-99 in river-based aquifer media recharged with reclaimed water. *Environ. Sci. Pollut. Res. Int.* 25 5140–5153. 10.1007/s11356-017-9143-x 28512710

[B23] Zhao S. Rogers M. J. Cao L. Ding C. He J. (2021). Identification of reductive dehalogenases that mediate complete debromination of penta- and tetra-brominated diphenyl ethers in *Dehalococcoides*. *Appl. Environ. Microbiol.* 87:em0060221. 10.1128/AEM.00602-21 34160266 PMC8357287

[B24] Zhao S. Rogers M. J. Ding C. He J. (2018). Reductive debromination of polybrominated diphenyl ethers – microbes, processes and dehalogenases. *Front. Microbiol.* 9:1292 10.3389/fmicb.2018.01292 29971048 PMC6018424

